# Supplementary material for: Degradation Kinetics and Shelf Life of N-acetylneuraminic Acid at Different pH Values
Source: Molecules. 2020 Nov 5;25(21):5141. doi: 10.3390/molecules25215141 (PMC7663827; doi:10.3390/molecules25215141)
Supplement: Supplementary file 1 [file molecules-25-05141-s001.pdf]

Supplementary materials

The analytical method validation

1. Verification of the accuracy of the method (sample recovery rate)

Take 8 samples, add the standard solution to the sample solution, and measure the recovery rate of the sample solution. As shown in Table S1, the recovery rate of the sample is between 99.3% and 100.4%.

Table S1. Sample recovery rate

Sample number	Concentration (g/L)	Theoretical concentration (g/L)	Recovery rate (%)
1#	0.2978	0.2981	99.9
2#	0.2950	0.2958	99.7
3#	0.3232	0.3241	99.7
4#	0.2981	0.2969	100.4
5#	0.2878	0.2898	99.3
6#	0.2746	0.2757	99.6
7#	0.2725	0.2746	99.2
8#	0.2842	0.2853	99.6

2. Verification of method precision

2.1 Instrument precision

Take the configuration sample (0.3g/L) compared with the standard (standard purity 99.2%), calculate the sample content, repeat 6 times, compare and analyze 6 groups of data results, evaluate the precision of the instrument according to RSD. At 99% confidence level, the confidence interval of the average value is 0.979 ±0.00255, 6 consecutive injections before and after injection, the contents are all in the confidence interval, and the precision of the evaluation instrument is RSD=0.15% according to the results of 6 groups of data.

Table S2. Instrument precision

Times	1	2	3	4	5	6
Sample peak area	3469813	3465661	3458679	3467779	3459845	3457607
Standard peak area	3509532.8					
Content	0.981	0.980	0.978	0.980	0.978	0.977
Average Content	0.979					
Confidence degree	0.99					
Confidence interval	0.979±0.00255（Upper limit 0.97645，Lower limit 0.98155）					
RSD%	0.15					

2.2 Precision within the method

Three samples (0.3g/L) were processed, each sample was configured for 3 times, and the sample was injected and analyzed for 3 times at the same time. The results of analysis data were compared, and the RSD of 3 times was calculated. The precision of the method was evaluated according to the RSD of 3 samples. According to the test results of three sets of data, the RSD of the first sample is 0.17%; the RSD of the second sample is 0.02%; the RSD of the third sample is 0.06%. According to the final three samples, the precision of the RSD evaluation method is 0.08%.

Table S3. Precision within the method

Times	1			2			3		
Sample number	1#-1	1#-2	1#-3	2#-1	2#-2	2#-3	3#-1	3#-2	3#-3
Sample peak area	3446200	3458431	3454436	3487791	3487277	3486438	3469813	3465661	3467779
Standard peak area	3583289	3583289	3583289	3569532	3569532	3569532	3509533	3509533	3509533
Content %	95.40	95.74	95.63	96.93	96.91	96.89	96.89	96.77	96.83
Average value	95.59			96.91			96.83		
RSD%	0.17			0.02			0.06		
Average RSD%	0.08								

2.3 Method reproducibility precision (different experimenters)

Three different experimenters were asked to configure the same sample as 0.3 g/L, performed both detection and analysis. According to the analysis of the data, the RSD of the first experimenter was 0.17%, the RSD of the second experimenter was 0.14%, and the RSD of the third experimenter was 0.12%. According to the RSD between the three experimenters, the precision of the reproducibility of the evaluation method is 0.025%.

Table S4. Precision of different experimenters

Experimenter	A			B			C		
Sample number	1#	2#	3#	4#	5#	6#	7#	8#	9#
Sample peak area	3418270	3429666	3431677	3405579	3400869	3406116	3437229	3420893	3434773
Standard peak area	3449741	3459232	3452010	3432289	3436986	3437307	3469803	3461272	3470561
Content %	98.30	98.35	98.62	98.43	98.16	98.30	98.27	98.04	98.18
Average value	98.42			98.30			98.16		
RSD%	0.17			0.14			0.12		
Average RSD%	0.025								

3. Speciality

The sample (0.3g/L) were processed, and the sample was injected and analyzed. The retention time of 8.513 is the peak of Neu5Ac, and the retention time of 6.443 is the peak of impurity.

The formula of resolution:

$$R=2(t_{R2}-t_{R1})/(W_1+W_2)$$

t_{R2}: Retention time of the latter peak of the two adjacent peaks;

t_{R1}: Retention time of the previous peak of the two adjacent peaks;

W_1、W_2: The peak width of the two adjacent peaks.

$$R=2(8.513-6.443)/1.172=3.532$$

It is stipulated in Chinese Pharmacopoeia that the resolution should be greater than 1.5, which proves that this method is with good specificity.

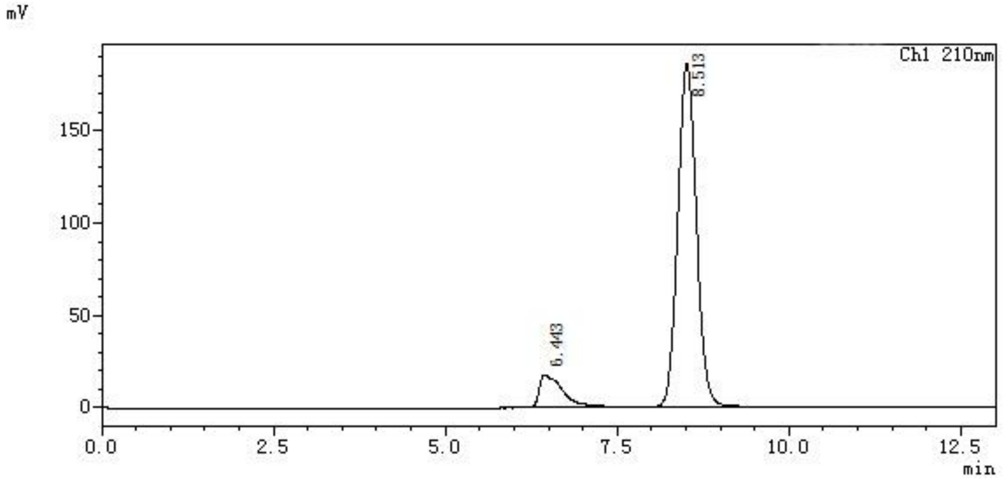

Figure S1. High performance liquid chromatography of Neu5Ac

4. Range

Range is the concentration range of the substance to be analyzed in the sample when certain accuracy, precision and linearity can be achieved.

The peak area of Neu5Ac within 5.0 g/L was detected. Then, taking the peak area as the ordinate (Y axis) and the concentration as the Abscissa (X axis), the curve of the relationship between concentration and peak area is drawn as shown in Figure S2. The specific verification method is to prepare 9 samples of test solution with different concentrations, determine the area of the peak respectively. According to the fact that the R ≥ 0.998, Y-axis intercept should be less than 2% of the 100% response value, and the relative standard deviation of the response factor is less than 2.0%, the linear range of the method is 0.1–0.5g/L.

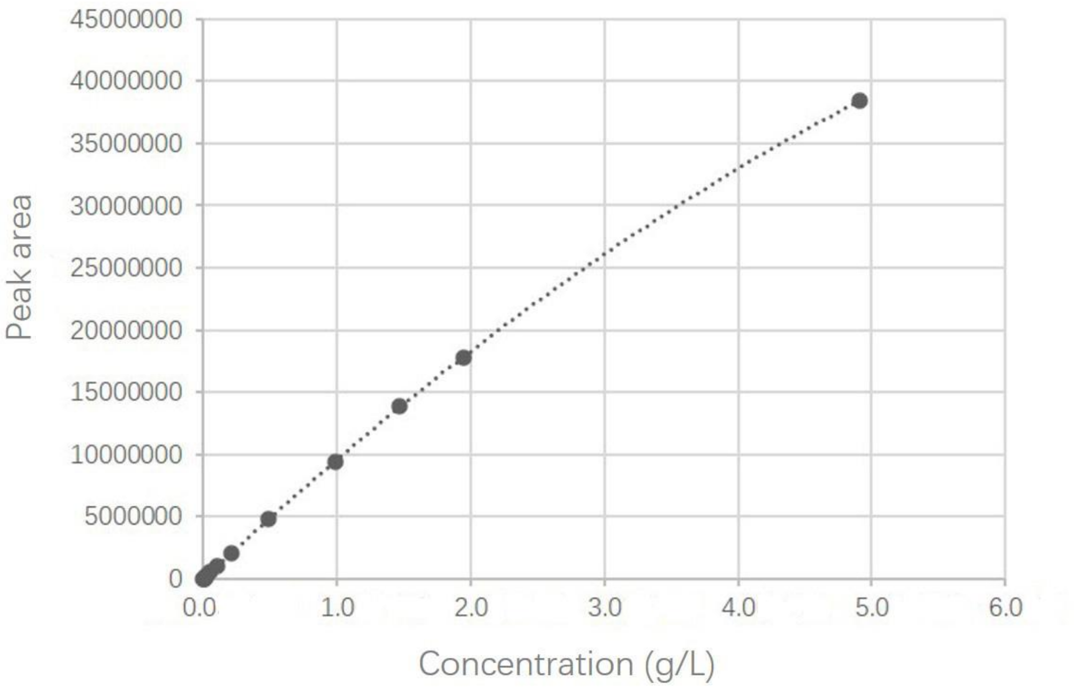

Figure S2. Relationship between Neu5Ac concentration and peak area

5. Linear verification

The sample (0.02g) was dissolved in 20 mL of distilled water， diluted the liquid to different multiples with distilled water . The samples were injected and analyzed. The test results of linear verification show that the detection method has a good linear relationship (R^2=0.9999).

The relative standard deviation of the corresponding factor is 0.989% which is less than 2% and the Y-axis intercept is 35724 which is less than 2% of the 100% response value.

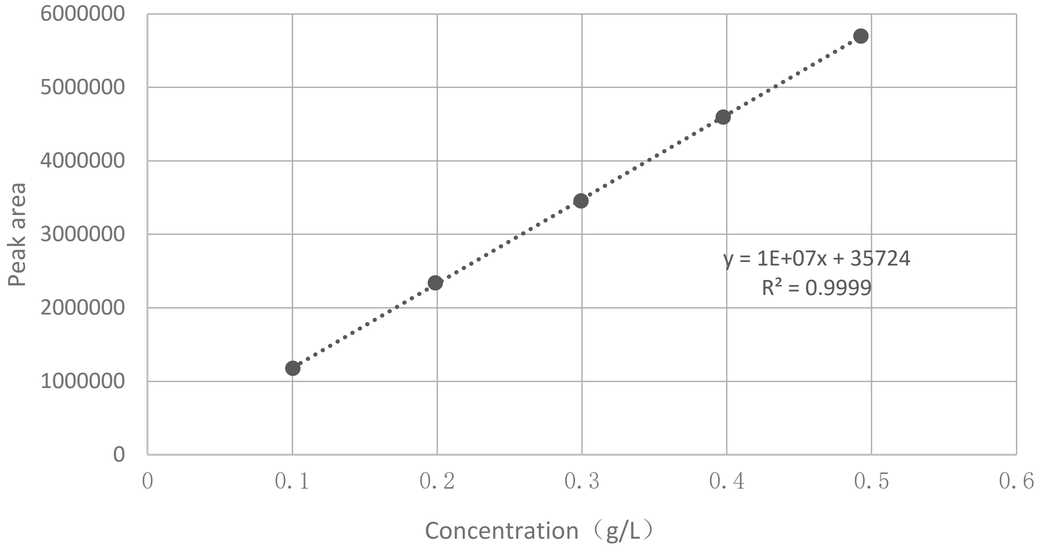

Figure S3. Linear verification

6. Limits of detection and limits of quantitation

The concentration of the sample is 0.3 g/L (purity 0.9835), and then diluted 10000 times as a parallel sample close to the blank.

The response values of the 20 parallel samples close to the blank are as follows:

Table S5. The response values of the 20 parallel samples

Number of samples	Response value (peak area)
20	550, 568, 527, 474, 572, 511, 595, 528, 506, 561, 558, 561, 554, 521, 562, 521, 573, 558, 555, 522

The average value is 543.85 and the standard deviation is 28.1747.

According to the formula:

$$C_L = K_i S_i \frac{C}{\bar{X}}$$

C_L: Minimum detectable concentration;
K_i: Confidence factor;
S_i : Standard deviation;
C: Sample concentration;
$\bar{X}$: Average of peak area

Under normal conditions:
(1) K_i =3
Limits of detection : 3×28.1747×0.3×0.9835×0.0001/543.85=4.59×10^{-6} g/L
(2) K_i =10
Limits of quantitation: 10×28.1747×0.3×0.9835×0.0001/543.85=1.53×10^{-5} g/L

The limits of detection is 4.59×10^{-6} g/L and the limits of quantitation is 1.53×10^{-5} g/L.
